# Early Life Trauma and Attachment: Immediate and Enduring Effects on Neurobehavioral and Stress Axis Development

**DOI:** 10.3389/fendo.2014.00033

**Published:** 2014-03-21

**Authors:** Millie Rincón-Cortés, Regina M. Sullivan

**Affiliations:** ^1^Department of Neuroscience and Physiology, Sackler Institute for Graduate Biomedical Sciences, New York University School of Medicine, New York, NY, USA; ^2^Emotional Brain Institute, Nathan Kline Institute for Psychiatric Research, New York, NY, USA; ^3^New York University Child Study Center, Department of Child and Adolescent Psychiatry, New York University School of Medicine, New York, NY, USA

**Keywords:** infant-attachment, maternal programming, development, amygdala, social behavior, rodent models, stress

## Abstract

Over half a century of converging clinical and animal research indicates that early life experiences induce enduring neuroplasticity of the HPA-axis and the developing brain. This experience-induced neuroplasticity is due to alterations in the frequency and intensity of stimulation of pups’ sensory systems (i.e., olfactory, somatosensory, gustatory) embedded in mother–infant interactions. This stimulation provides “hidden regulators” of pups’ behavioral, physiological, and neural responses that have both immediate and enduring consequences, including those involving the stress response. While variation in stimulation can produce individual differences and adaptive behaviors, pathological early life experiences can induce maladaptive behaviors, initiate a pathway to pathology, and increase risk for later-life psychopathologies, such as mood and affective disorders, suggesting that infant-attachment relationships program later-life neurobehavioral function. Recent evidence suggests that the effects of maternal presence or absence during this sensory stimulation provide a major modulatory role in neural and endocrine system responses, which have minimal impact on pups’ immediate neurobehavior but a robust impact on neurobehavioral development. This concept is reviewed here using two complementary rodent models of infant trauma within attachment: infant paired-odor-shock conditioning (mimicking maternal odor attachment learning) and rearing with an abusive mother that converge in producing a similar behavioral phenotype in later-life including depressive-like behavior as well as disrupted HPA-axis and amygdala function. The importance of maternal social presence on pups’ immediate and enduring brain and behavior suggests unique processing of sensory stimuli in early life that could provide insight into the development of novel strategies for prevention and therapeutic interventions for trauma experienced with the abusive caregiver.

## Introduction

Both animal and human research demonstrate that early life experiences interact with genetics to program the central nervous and endocrine systems, including the hypothalamus–pituitary–adrenal (HPA)-axis (1–5). Infant experiences typically occur within the context of the mother and the quality of caregiving by the mother, determined by the patterning and intensity of maternal stimulation of pups’ sensory systems, is a key regulator of HPA-axis neuroplasticity in the neonatal period (6–10). Dissecting the mother–infant dyad has characterized maternal control over infant brain and behavior through “hidden regulators” present during mother–infant interactions (11, 12). Lack or loss of typical parental stimulation is a potent stressor during early life (13, 14), and removal of these hidden regulators through maternal deprivation, modulation of maternal behavior, and/or traumatic interactions with the mother, produce immediate changes in pups and result in wide-spread dysregulation of physiological and behavioral responses during development (15–26). Within the range of typical parenting, normal variations in maternal care during infancy program individual differences in behavioral and endocrine responses to stress in rodents and humans; although pathological experiences, including abuse and neglect, produce vulnerability to later-life psychiatric disorders (7, 27–37).

Here, we focus on infant experiences and the effects of early life stress and HPA-axis activation as experienced within the mother–infant dyad, as well as the pups’ attachment to the caregiver and learning about the caregiver. We review two complementary rodent models of infant trauma within attachment: infant paired-odor-shock conditioning and rearing with an abusive mother, which converge in producing a similar neurobehavioral phenotype in later-life consisting of depressive-like behavior as well as disrupted HPA-axis and amygdala function, thus enabling us to explore both the immediate and enduring effects of abusive attachment as well as role of the HPA-axis and the stress hormone corticosterone (CORT). Although infant trauma resulting from abusive attachment affects neural substrates of stress vulnerability and resilience, these can be engaged by sensory cues learned during infancy (i.e., artificial or natural maternal odor), which have the ability to normalize adult neurobehavioral dysregulation stemming from early life trauma.

### Attachment

Attachment is a psychosocial process referring to the deep and enduring emotional bond that connects two individuals across space and time, with an individual deriving security from physical and psychological contact with the attachment figure (38–40). Attachment requires experience-dependent learning of the sensory stimuli associated with infant–caregiver interactions, and a strong attachment to the caregiver is crucial for survival in altricial species, including humans (41–48). In children, attachment is characterized by specific behaviors such as seeking proximity to the caregiver, whom provides a sense of safety and security for the infant (49–51). Like humans, infants from altricial species also exhibit attachment related behaviors to their caregiver shortly after birth that elicit nurturing and attachment from the caregiver, which entails responding appropriately to the infant’s needs by providing nourishment, protection, and warmth necessary for survival (51–53). Thus, infant-attachment is an adaptive and reciprocal process consisting of a dynamic and complex exchange of mother–infant behavioral interactions that enhance the infant’s chance of survival by maintaining contact with the caregiver.

The mother–infant attachment bond is among the strongest social attachments formed by most mammals (54). As such, human infants seek proximity to and maintain contact with the caregiver despite the quality of care they receive (55), including attachment to an abusive caregiver. This paradoxical phenomenon also occurs in dogs, chicks, and non-human primates, suggesting a phylogenetically preserved system (32, 41, 43, 56–63). From an evolutionary perspective, attachment to an abusive caregiver is thought to be adaptive because it provides immediate benefits, as the infant still has access to some care (48, 64). Albeit infant organisms are biologically predisposed to attach to their caregiver and possess behavioral systems that allow them to rely on these bonds for survival (38), clinical and preclinical studies suggest that adverse parental care compromises brain development and has longstanding effects in stress-responsive neurobiological systems, including the HPA-axis, neurotransmitter systems, as well as cortical and limbic structures such as the prefrontal cortex, amygdala, and hippocampus (65–75). Moreover, traumatic early life experiences involving the caregiver increase the risk for a wide-range of deleterious mental health and behavioral outcomes, including developmental psychopathology, affective, and mood disorders (37, 72, 76–86). Therefore, perturbations in infant-attachment appear to induce immediate neurobiological changes that shape subsequent development and lead to neurobehavioral dysregulation associated with compromised emotionality and increased vulnerability to psychopathology during later-life, suggesting that the quality of an infant’s first social relationships programs the infant’s emotional and cognitive capabilities to adapt to later-life environments.

Despite the fact that childhood abuse remains a major public health concern (87–91), the mechanisms by which infant trauma initiates the pathway to psychopathology are poorly understood, although the stress axis is evidently implicated. However, animal models have provided some insight into the mechanisms by which disruptions in parental care alter the development of stress response systems (92, 93), which may contribute to our understanding of resilience following infant trauma (62, 94–98). For example, research using animal models of maternal deprivation in rodents and non-human primates parallel human imaging studies suggesting that disruptions in infant-attachment also produce long-term alterations in the limbic system and the stress axis that may compromise the development of emotion- and attention-regulatory systems, which has been used to explain the heightened risk of behavioral and affective disorders in human children experiencing adverse parental care (13, 31, 32, 75, 93, 99–108). Overall, these studies demonstrate that parental care affects the maturation of these brain areas and offers potential sites to understand the damaging effects of early life abuse on subsequent neurobehavioral development (31, 70, 71, 74, 84, 94, 109–115). For these reasons, we employ rodent models of abusive attachment and study the infant’s immediate response to trauma as well as the neurobiological sequelae leading to later-life neurobehavioral dysregulation to better understand the infant mechanisms that initiate the pathway to later-life psychopathologies.

### The stress-hyporesponsive period and maternal regulation of the HPA-axis

In rats, infant-attachment occurs within a unique developmental context – the stress-hyporesponsive period (SHRP) – during which neonates show low basal plasma concentrations of CORT and reduced stress-reactivity, as indexed by limited adrenocorticotropic hormone (ACTH) and CORT responses to stressful stimuli compared to older animals, as well as low levels of corticosteroid binding globulin (CBG), which regulates glucocorticoid (GC) access into the brain (92, 116–123). Thus, the neuroendocrine stress response of the neonatal rat is characterized by attenuated hormonal responses and altered gene regulation in response to stress compared to adults due to hyporesponsiveness at all levels of the HPA-axis, namely: (1) a blunted pituitary ACTH secretion, resulting from a combination of immaturity of neural inputs to the corticotropin releasing hormone (CRH) neurons, (2) decreased pituitary peptide content or decreased sensitivity to CRH stimulus; and (3) an adrenal gland hyporesponsive to circulating ACTH levels (18, 119, 121, 124–130). Accumulating evidence suggests that human infants exhibit a period of dampened cortisol reactivity analogous to the rodent SHRP, which develops gradually over the course of the first year of life (~6–12 months), although it remains unclear how long it extends (131–135). In both humans and rodents, the SHRP is thought to protect the developing brain from the detrimental effects of elevated HPA-axis activity and excess GC exposure, and the sensitivity and responsiveness from the caregiver appears critical in maintaining low cortisol activity and controlling the offspring’s physiological and behavioral responses to stressors during this period (3, 30, 32, 68, 100, 122, 127, 129, 136–140).

However, the SHRP during development appears to be stressor specific, since the HPA-axis is fully capable of responding to stimuli that may be considered stressful to a neonatal rat such as cold or saline injection (141–145). Indeed, the HPA-axis and CORT receptors are functional at birth, but are modulated by the sensory stimulation provided by the mother (100, 119, 126, 146–152). Moreover, the mother is able to directly regulate the pups’ CORT levels through hidden regulators embedded in typical mother–infant interactions, such as the sensory, motor, nutrient, and thermal events associated with caregiving, which exert regulatory influence over the infant’s immediate and long-term behavioral and physiological responses by affecting sleep-wake states, cardiac rates, and HPA-axis function (6, 10–12, 17, 129, 153, 154). Removal of maternal sensory stimulation during the SHRP, such as that occurring when the pups are separated from the mother for a prolonged period of time (i.e., maternal deprivation paradigm), increases CORT secretion (16), elevates CORT levels in pups (11, 12, 129), and enables higher CORT/ACTH responses to acute stress (15, 19, 100, 145, 155). Importantly, these changes are similar to those induced by normal variations in maternal care (i.e., maternal high/low licking paradigm) (7, 27, 29) as well as atypical or abusive maternal care (20, 144, 156), suggesting that the hypothalamic mechanisms controlling physiological stress responses in the pup are regulated by elements of maternal care. Taken together, these findings suggest that maternal deprivation, variations in maternal care, and abusive maternal care influence the development and function of the HPA-axis (8, 9, 30, 112, 114, 157–160). In summary, maternal stimulation modulates the infant’s HPA-axis and maintains the SHRP, although potent stressors involving disruptions in maternal stimulation (i.e., cold, maternal deprivation, atypical maternal care) can activate the HPA-axis and override maternal control of the SHRP.

## Attachment Learning during a Sensitive-Period in Rat Pups

**Figure 1:**
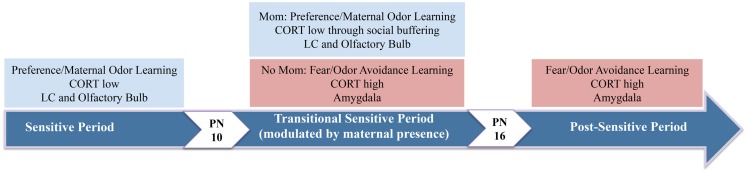
**The neural circuitry underlying pup attachment learning changes over development**. During the earliest days of life, pups have a sensitive-period in which odor-shock conditioning produces an odor preference. At 10 days of age, pups begin the transitional sensitive-period, when pups endogenous CORT levels have increased sufficiently to enable amygdala-dependent fear/avoidance learning. However, with the mother present at this age, pups will revert back to preference learning and the neural circuitry of the sensitive-period. Thus, the mother’s presence socially buffers pups (i.e., attenuates pups shock-induced CORT release) and pups learn a preference. As pups mature and enter the post-sensitive-period, odor-shock conditioning induces amygdala-dependent fear and odor avoidance learning (25, 165–167).

Infants possess a predisposition to approach the mother as well as specific sensory cues associated with her care, such as her odor and vocalizations (161, 162). Within an evolutionary context, the infant-attachment system serves to establish a preference for the mother regardless of whether or not she is associated with pain or pleasure (48, 64). This type of survival-dependent learning is known as imprinting, has wide phylogenetic representation, and is temporally confined to a sensitive-period in development (50, 161, 163) typically involving a hypofunctioning HPA-axis – the principal pathway of the mammalian stress response that regulates the production of GCs (cortisol in humans, CORT in rodents) (40, 164). In rats, we refer to this period of enhanced attachment/preference learning as the “sensitive-period,” or postnatal (PN) days 1–9 (see Figure 1). As we will discuss below, sensitive-period learning is due to the pup’s unique learning circuit, presumably one sculpted through evolution to provide infants with the neural circuitry required to survive and maximize attachment to a caregiver (48).

Intriguingly, the sensitive-period for attachment learning in rat pups overlaps with the SHRP, suggesting that low levels of CORT and reduced HPA-axis responsiveness may contribute to the neonate’s unique neural circuitry for attachment learning. However, in order for infant-attachment to occur, the rat pup must first learn to identify the caregiver and exhibit the social behaviors necessary for survival such as orienting to and approaching the caregiver, grasping the nipple and nursing (50, 168, 169). Infant-attachment learning in rodents revolves around the pup’s ability to learn and develop a preference for the mother’s odor, which is diet dependent and can change postnatally (47, 170–174). Since rat pups are born deaf and blind, they must rapidly learn their mother’s odor, which conveys distal and proximal information about the mother’s location, and helps the pups orient to the mother, approach her and elicit care (169, 175). The maternal odor is critical in guiding infant-attachment; without it, pups show reduced contact with the mother, are unable to nipple attach and exhibit low survival rates (25, 176). Moreover, any neutral odor can acquire properties of the natural maternal odor and act as a new maternal odor by simply being placed on the mother, in a cage during mother-infant interactions (177–182) or learned in classical conditioning paradigms (i.e., odor-stroke, odor-shock) performed outside the nest in the absence of the mother (111, 165, 171, 183–188).

Our lab uses infant olfactory classical conditioning in which an artificial odor (i.e., peppermint) is paired with a 0.5 mA shock as a rodent model of abusive attachment. While the adult rat responds to shock with a robust CORT response, the neonatal rat does not (9, 100, 189). Unlike older animals, which readily learn odor aversions to painful stimuli paired with an odor, rat pups actually exhibit an odor preference and approach the odor (111, 165, 190–193). This odor preference, however, is not due to the inability of pups to feel pain, since the pain threshold varies little during the neonatal period and pups emit vocalizations to the shock, suggesting that they are experiencing distress (165, 190, 194–197). Instead, infant paired-odor-shock conditioning produces a new artificial maternal odor that acquires the ability to regulate pup behaviors typically controlled by the maternal odor; it induces proximity-seeking/approach responses in pups (distal cues), guides mother–infant interactions by facilitating contact with the mother and nipple attachment (proximal cues), and activates the same neural circuitry as the natural maternal odor (25, 111, 165, 169), suggesting that this odor has comparable qualities to the natural maternal odor. Importantly, infant odor-shock conditioning is a useful experimental paradigm for understanding how early life trauma (i.e., pain-shock) can support and maintain attachment and provide insights into the particular ways the infant brain processes painful stimuli and its relationship to the enduring effects of this experience due to the well documented neural circuitry underlying this type of learning (198–202). Three brain structures have been shown to play a role in the neonatal rat’s sensitive-period for enhanced odor learning: the olfactory bulb (OB), the noradrenergic locus coeruleus (LC), and the amygdala (50, 203).

## Neurobiology of Infant-Attachment and the Role of the HPA-Axis in Terminating Attachment Learning

Neonatal odor learning produces changes in the OB, which can be induced both naturally in the nest and experimentally in controlled learning experiments outside the nest (182, 186, 204–210). For example, both natural and learned odors produce a similar enhancement of OB responding during the sensitive-period, which has been assessed through a variety of techniques including 2-deoxy-glucose (2-DG) uptake, c-Fos immunoreactivity (ir), CREB phosphorylation, electrophysiology, and optical imaging (205–208, 211–214). Thus, olfactory-based attachment learning in neonatal rats is associated with the acquisition of odor-specific neural changes in the OB, which can only be acquired during the sensitive-period, and are retained throughout development (111, 201, 215–218).

Infant rats (PN1–9) readily learn an odor preference to neutral odors paired with pleasant (i.e., milk, stroking) (47, 171, 179, 183–185, 219) or painful stimuli, such as 0.5 mA shock or tail pinch (111, 165, 190, 191, 201), which is partly due to a uniquely large noradrenergic input to the OB from the LC, the sole source of norepinephrine (NE) for the OB (220, 221), which prompts abundant release of NE into the OB (203, 222). Furthermore, the neonatal LC shows prolonged stimulus-evoked excitation and greater NE release to odors during the sensitive-period compared to later-life due to the immaturity of the LC alpha-2 inhibitory autoreceptors, which functionally emerge around PN10 and cause a shift from prolonged excitatory alpha-1 mediated responses to inhibitory alpha-2 mediated responses, resulting in brief excitation due to inhibited LC firing and decreased NE output (203, 222–227). Importantly, NE release from the LC is both necessary and sufficient for odor preference learning during the sensitive-period (228–232).

Experimental evidence indicates a lack of amygdala participation in the neural circuitry underlying infant paired-odor-shock conditioning during the sensitive-period, as suggested by amygdala lesions, 2-DG, and c-Fos-ir (111, 201, 203, 216, 232), although the amygdala is strongly implicated in adult classical conditioning (198–200, 202, 233). These data suggest that the infant amygdala is not part of the sensitive-period learning circuit during which aversions are difficult to learn because of its failure to exhibit the plasticity required for this type of learning (234–236), although the amygdala is responsive to odors and other environmental stimuli by PN10 (165, 201, 237). Like the infant amygdala, the infant HPA-axis is limited in function, resulting in reduced shock-induced CORT release during the neonatal sensitive-period (189), which limits pups’ ability to acquire learned odor aversions (201, 238). Endogenous CORT levels increase gradually and reach a critical level by PN10 (92, 136, 239, 240), at which time stressful or painful stimuli are able to elicit a sufficient CORT response that permits infant amygdala plasticity and avoidance learning (Figure 1) (201, 218, 241).

Indeed, the natural increase of stress-induced CORT release marks the end of sensitive-period learning (165, 201, 203, 238), which has been demonstrated experimentally by increasing CORT systemically (3 mg/kg, i.p.) or through intra-amygdala CORT infusions (50–100 ng) prior to odor-shock conditioning, which enables sensitive-period pups to learn an odor aversion and exhibit learning-evoked neural activity (i.e., enhanced 2-DG uptake) in the amygdala, while preventing the acquisition of learning-induced changes in the OB (201, 238, 241, 242). In contrast, CORT depletion (via adrenalectomy or social buffering, discussed below) in PN12 pups results in shock-induced odor preference learning and acquisition of OB neural changes. Thus, within the context of paired-odor-shock conditioning, CORT appears to play a modulatory role on infant learning by switching whether the amygdala learns attraction or avoidance: if CORT is low, pups learn a preference to an odor paired with shock due to a lack of amygdala involvement; if CORT is high, the amygdala is activated by odor-shock conditioning and pups learn an avoidance. Recently, we have identified a role for amygdala dopamine (DA) in mediating these infant learning transitions, as conditions that block aversion/fear learning are associated with downregulated DA function (243). Altogether, these findings suggest that neonatal rat pups have unique learning capabilities that aid olfactory-based attachment to the mother, which are dependent on low levels of CORT.

In summary, the infant learning circuit is characterized by an enhanced ability to learn odor preferences to aversive stimuli, due to a hyper-functioning LC, as well as a decreased ability to learn odor aversions that may interfere with proximity-seeking during the sensitive-period due to a hypo-functional amygdala, suggesting that the infant brain is specialized for maximizing attachment to a caregiver (Figure 1) (41, 165, 186, 221, 225, 229, 234, 235, 244–247). As the sensitive-period ends, owing to the natural emergence of CORT, odor aversions can be learned because of changes in the infant learning circuit, including maturation of LC autoinhibition, which reduces NE release and greatly attenuates rapid odor preference learning, but also due to the functional emergence of the amygdala, all of which enable the plasticity required for aversion learning (50, 165, 223, 225, 229, 232, 241).

## Maternal Modulation of HPA-Axis Function and Sensitive-Period Duration

Empirical evidence suggests that social support is a powerful modulator of individual differences in response to potentially stressful events in both humans and animals (248–253). In rodents, maternal presence is known to blunt CORT release to stressful and painful stimuli in older pups (>PN12) through olfactory and somatosensory cues (9, 148, 152, 166, 167, 254, 255). The process by which the presence of a social companion and/or social sensory cues can dampen HPA responses to stressors (i.e., decrease CORT levels) is termed “social buffering” and has been reported in humans and other species (139, 249, 250, 253, 256–259). Our lab has identified a transitional sensitive-period in pups from PN10–15, during which odor-shock conditioning produces either olfactory preference or aversion in infant rats depending on social context (166, 260). In the absence of the mother, paired-odor-shock conditioning yields a learned odor avoidance that is accompanied by amygdala activation. However, maternal presence is able to suppress amygdala activity and block aversion learning induced by odor-shock conditioning, indicating that maternal presence reengages the sensitive-period attachment circuitry to reinstate odor preference learning through modulation of CORT (see Figure 1), and therefore CORT regulation of amygdala activity. Importantly, these animal data are consistent with the principles of attachment theory (38), in which access to a secure base provided by the attachment figure reduces the probability of HPA/CRF stress reactions that could have unfavorable long-term consequences on brain development (9, 137, 261).

Yet, human parental care is disturbed under conditions of chronic stress (262), which can be modeled in rodents by creating an abnormal rearing environment that alters maternal behavior (20, 23, 111) and mimics the effects of a stressful environment as a risk factor for potentiating infant abuse, including humans (62, 77, 263, 264). Because bedding type and volume are important components of the dam’s nesting environment, limiting the amount of bedding available constitutes a continuous stressor for the dam and her pups, disrupts mother–pup interactions, and alters the development of the pup’s HPA-axis by reducing the frequency of positive maternal behaviors (i.e., licking, grooming, nursing) and increasing the frequency of negative maternal behaviors that are painful to the pup and elicit vocalizations, such as stepping, dragging, and rough handling of the pups (20, 25, 111, 156, 188, 265). Thus, one could conceptualize a stressed dam as a poor regulator, which is supported by findings showing that ICV infusion of corticotropin releasing factor (CRF) reduces maternal responsivity (266).

Furthermore, because maternal stimulation of pups modulates pups’ endogenous CORT, maternal care quality alters sensitive-period duration. Pups reared with a stressed mother (i.e., poor regulator) exhibit a precocious emergence of CORT, which is delivered through the mother’s milk (267), that facilitates aversion learning and engages the amygdala, as indexed by increased odor-shock-induced amygdala neural activity (188), suggesting that experience with a stressed mother prematurely ends the SHRP and the sensitive-period for attachment learning. In addition, this procedure results in striking changes in the expression and activity patterns of key regulatory elements of the neuroendocrine stress response, which result in persistent alterations of HPA-axis function such as elevated basal GC concentrations, impaired GC feedback, and modifications in CRF-receptor regulation (20, 25, 114, 156, 174). Since the mother serves as a primary link between the environment and the infant, environmentally driven alterations in maternal care could transduce an environmental signal to the pups, alter the development of central CRF systems activating behavioral, endocrine and autonomic responses to stress, as well as systems regulating CRF and HPA-axis activity, which may serve to increase or decrease stress-reactivity in the offspring, so that it mirrors that of the mother.

## Immediate and Enduring Effects of Early Life Stress

Responses to stressors, or conditions that threaten or are perceived to threaten physiological equilibrium, are mediated by the activation of stress-responsive neurobiological systems that help preserve allostasis, or stability through change, thereby making the stress response an essential endocrine mechanism for survival (268–270). Stressors, which can include psychological and physical challenges, increase the amount of hypothalamic CRF that is released into the anterior pituitary gland, stimulating ACTH secretion in the anterior pituitary and resulting in GC production in the adrenal gland (268, 271, 272). GCs facilitate the mobilization of substrates for energy sources, potentiate the release of catecholamines, and enhance cardiovascular tone while suppressing “non-essential systems” for immediate survival, such as immunity, growth, and reproduction (273–276). Stress-induced HPA-axis activation is associated with acute release of stress-related neuropeptides, hormones, and neurotransmitters, including NE, serotonin (5-HT), and DA, in cortical and limbic structures (21, 27, 277–289). Although acutely elevated GCs help orchestrate physiological and behavioral responses that promote allostasis, chronic activation of the HPA-axis, and prolonged elevations of GCs and CRF increase the risk of stress-related disorders and psychological illnesses during later-life (269, 290–292).

The effects of HPA-axis activation depend on multiple factors, including the developmental stage in which the insult occurs, number of exposures, and type of adversity (71, 293–297). Numerous behavioral, endocrine, and clinical studies have shown that various early life stressors cause a premature increase in CORT levels (129) that produces profound alterations in growth and development and negatively affects mental health (40, 72, 135, 298, 299). Moreover, repeated exposure to early life stressors, both physical and psychological, induce changes in endocrine (HPA-axis), neurotransmitter (DA, 5-HT), and brain memory systems, including the hippocampus, amygdala, and PFC that persist throughout the life-span (8, 67, 101, 300, 301). Furthermore, the HPA-axis is modulated by limbic and cortical regions such as the amygdala, hippocampus, and the PFC (269, 302), which enable the activation of stress responses by psychosocial stressors (303–307). Importantly, the timing of early life stress may affect brain regions undergoing specific growth spurts during that time (308, 309), so that brain regions rich in GC receptors and characterized by extended PN development, such as the amygdala, hippocampus, and PFC, are particularly susceptible to the long-term effects of stress (71, 92), which affects later-life memory, cognitive, executive, and affective function as well as stress-reactivity in humans (296, 297). Alterations in stress-sensitive neurobiological systems, including regulation of GCs and CRF, have been posited as mechanisms through which early life stress, including inadequate/disorganized parental care, increases the likelihood of psychopathology by influencing HPA hyperreactivity to stressors and promoting the development of stress-induced illnesses throughout life (31, 40, 290, 310–312).

Early life adversity may lead to a maladaptive outcome to a given later environmental context. Depression is a common outcome of childhood abuse, and children with a comorbid history of depression and abuse have elevated CRF levels in the cerebrospinal fluid (313) as well as an increased ACTH response to a CRF challenge compared to children with depression without abuse, suggesting excessive CRF release (3, 314, 315). Additional clinical evidence indicates that severe early life stressors in childhood are associated with the long-term HPA-axis disturbances in depressed patients (316–319), which is supported by preclinical studies of non-human primates showing that poor rearing conditions and conditions that disrupt responsive maternal care have a long-term impact on the neurobiology of stress and negative emotionality (21, 31, 32, 109, 158). For example, variable foraging paradigms that result in neglectful maternal care produce adult offspring that are more fearful, low in dominance, have elevated levels of CRF in the CSF and high in brain levels of CRH, exhibit persistent alterations in metabolites of 5-HT, DA, and NE, as well as changes in noradrenergic and serotonergic responses to stress (99, 320–324). Given the importance of noradrenergic and serotonergic systems in mood disorders, these findings postulate a mechanism by which early life stress may predispose an individual to later-life depression (32, 300, 325–327).

## Convergence of Both Abusive Attachment Models in Producing a Depressive-Like Behavioral Phenotype during Later-Life

Recently, our lab has demonstrated that both rodent models of abusive attachment (paired-odor-shock, abusive mother) during infancy result in later-life depressive-like behavior in the Forced Swim Test (FST), a measure of behavioral despair in rodents (328, 329), that is accompanied by changes in amygdala function and preceded by disruptions in social behavior (26). When employed from PN8–12, these two complementary rodent models of early life abuse produced a reduction in sociability, as indexed by spending significantly less time in a social chamber compared to control animals reared with a normal mother – a behavioral pattern that was observable prior to weaning (PN23) and maintained in adolescence (PN45). However, animals experiencing early life abuse only showed depressive-like behavior in the FST during adolescence (PN45), as indicated by immobility – the passive state in which the animal makes only those movements necessary to keep its head above water (328, 330). In addition, depressive-like behavior in the FST in animals experiencing early life abuse was associated with increased c-Fos-ir in the basal, lateral, and central amygdala nuclei, suggesting that increased neural activity in these structures may contribute to the expression of depressive-like behavior in the FST (26). A causal relationship between amygdala function and depressive-like behavior in the FST was suggested through temporary inactivation (i.e., muscimol) of amygdala function during the FST, which normalized these behaviors to a level comparable to controls (26). Collectively, these findings suggest that the expression of depressive-like behavior in the FST following early life abuse is characterized by a hyper-functioning amygdala. Thus, abusive attachment appears to disrupt the developmental trajectory of the amygdala and modify the way that it responds to future stressors, which is supported by our work using rodent models of early life abuse.

Our findings are in accordance with clinical and animal literature indicating that early life adversity constitutes a prime risk factor for the development of psychopathologies characterized by dysregulated HPA-axis function (1, 5, 24, 32, 133, 319, 331–334), such as mood and affective disorders (37, 93, 335, 336), which also exhibit a developmental delay (309, 337, 338) Thus, these rodent models of early life abuse allow us to explore the ontogeny of depressive-like behavior and amygdala dysregulation, which is of clinical relevance because abnormal amygdala function and social behavior deficits as well as their relationship to later-life depressive-like behaviors have been documented in individuals with a history of early life abuse (71, 310, 331, 336).

## Modulation of Adult Neurobehavioral Function by Infant-Attachment Related Cues

An ample body of evidence suggests that the quality of infant-attachment relationships results in long-term adaptations that have the ability to program subsequent behavioral, endocrine, and neural function (28, 109, 261, 310, 336). Results from our laboratory have shown that infant paired-odor-shock conditioning results in reduced fear learning and attenuated related amygdala function, dysregulation in neural networks underlying olfactory learning, and depressive-like behavior during adulthood (339–342). Importantly, attachment related sensory cues learned during infancy can play a critical role in modulating neurobehavioral responses during later-life. In humans, for example, cues associated with early life abuse elicit strong attraction and feelings of comfort (343). In rodents, presentation of an artificial maternal odor, resulting from infant paired-odor-shock conditioning, is able to reverse the behavioral effects of abusive attachment in rodent measures of depressive-like behavior, such as the sucrose consumption test and the FST (342). Specifically, the odor increased the latency to immobility and reduced the time spent immobile in the FST, but also increased the percentage of sucrose consumed during a sucrose preference test to levels comparable to controls. Furthermore, these restorative effects of a learned infant maternal odor on adult function were also observable at electrophysiological level, as odor presentation also normalized paired-pulse inhibition deficits in the amygdala. Collectively, these data suggest that early life experiences are able to shape adult neural circuits underlying behavior and that adult behaviors can be modified under environmental conditions in which learned infant cues are present. The discovery that infant cues can retain their value throughout the life-span and regulate later-life behaviors controlled by circuits implicated in emotion, learning, and social behavior is of great interest because it provides an opportunity for intervention and possibly correction of maladaptive outcomes related to psychopathology induced by adverse early life experiences within attachment. Thus, it appears that the enduring neurobehavioral dysregulation stemming from early life abuse can be positively modulated by learned sensory cues related to infant-attachment.

## Conclusion

In species requiring parental care, evolution has ensured that infants quickly learn and express robust preferences to the caregiver, regardless of the quality of care (48, 50). However, trauma within attachment leaves the infant particularly vulnerable to adult psychiatric disorders, behavioral changes in fear and anxiety, and alterations in neural circuits, particularly those regulating stress and emotion (71, 133, 334, 344, 345). In addition, early life stress can have negative effects on the neurobiology of the developing brain that are comparable to those induced by disruptions in infant-caregiver interactions (25, 346) Thus, early life experiences have enduring effects on the neuroplasticity of the HPA-axis, suggesting the HPA-axis is programmable via multiple environmental sources across development. In early development, stressors and maternal care jointly program HPA-axis responses and later-life function. The HPA-axis, however, remains modifiable during later stages of development during which infant-attachment related cues can exert a positive modulatory effect on later-life HPA-axis function as well as behavioral and endocrine responses to stress.

## Conflict of Interest Statement

The authors declare that the research was conducted in the absence of any commercial or financial relationships that could be construed as a potential conflict of interest.
